# Are Drug Companies Living Up to Their Human Rights Responsibilities? The Merck Perspective

**DOI:** 10.1371/journal.pmed.1000343

**Published:** 2010-09-28

**Authors:** Geralyn S. Ritter

**Affiliations:** Merck & Co., Whitehouse Station, New Jersey, United States of America

## Abstract

The human rights responsibilities of drug companies have been considered for years by nongovernmental organizations, but were most sharply defined in a report by the UN Special Rapporteur on the right to health, submitted to the United Nations General Assembly in August 2008. The “Human Rights Guidelines for Pharmaceutical Companies in relation to Access to Medicines” include responsibilities for transparency, management, monitoring and accountability, pricing, and ethical marketing, and against lobbying for more protection in intellectual property laws, applying for patents for trivial modifications of existing medicines, inappropriate drug promotion, and excessive pricing. Two years after the release of the Guidelines, the *PLoS Medicine* Debate asks whether drug companies are living up to their human rights responsibilities. Sofia Gruskin and Zyde Raad from the Harvard School of Public Health say more assessment is needed of such responsibilities; Geralyn Ritter, Vice President of Global Public Policy and Corporate Responsibility at Merck & Co. argues that multiple stakeholders could do more to help States deliver the right to health; and Paul Hunt and Rajat Khosla introduce Mr. Hunt's work as the UN Special Rapporteur on the right to the highest attainable standard of health, regarding the human rights responsibilities of pharmaceutical companies and access to medicines.

## Abstract 2

As one viewpoint of three in the *PLoS Medicine* Debate on whether drug companies are living up to their human rights responsibilities, Geralyn Ritter, Vice President of Global Health Policy and Corporate Social Responsibility at Merck & Co., argues that that multiple stakeholders could do more to help States deliver the right to health.

*This is the second of three viewpoints examining the question of whether pharmaceutical companies are living up to their human rights responsibilities.*

## 

As a global health care company, Merck believes that helping to address global health challenges is a strategic and humanitarian imperative. While States bear primary responsibility for the realization of the right to health, it requires a genuine effort among many actors, including governments, multilateral organizations, nongovernmental organizations, health care professionals, and the pharmaceutical industry. This collaboration is particularly important given the many complex factors that adversely affect the right to health, such as poverty, poor infrastructure and distribution channels, corruption, lack of health care education and awareness, inadequate public health services, and lack of adequately trained health care professionals.

## The Role of the Pharmaceutical Industry in Relation to the Right to Health

The pharmaceutical industry can and should leverage its expertise to help States achieve the full realization of the right to health and should work in partnership with them and other actors to help remove barriers that stand between patients and the health care they need. It is the right thing to do not only from a moral perspective, but from a long-term commercial perspective as well.

The primary role of the pharmaceutical industry in realizing the right to health is through its core capabilities of researching, developing, and producing medicines and vaccines to address unmet medical needs, and in helping to ensure their appropriate distribution. In this role over the past century, the industry has helped to save or improve the quality of millions of lives. For example, the industry's role in developing and distributing vaccines for diseases such as measles has supported the near elimination of that disease in many regions of the world [1]. Pharmaceutical companies have also created and delivered antiretrovirals that have saved millions of lives in the global fight against HIV/AIDS [2].

In fulfilling this primary role, the industry must of course act in a responsible manner – protecting employee safety, working to reduce its environmental impact in local communities, and safeguarding the safety and privacy of clinical trial participants. Membership of many companies in the UN Global Compact reflects this commitment to responsible behavior.

The role of the pharmaceutical industry in promoting access to medicines has received particular attention. In recent years, pharmaceutical companies have strengthened their focus on ways they can support and promote access to health, especially in developing countries, where the needs and challenges are so significant. Areas where the pharmaceutical industry can play an important role, working with States and other partners, include the following:

- ***Discovering and developing new medicines for neglected diseases prevalent in developing countries*** **.** In 2008, 67 company-backed projects targeting the ten priority “neglected” diseases were ongoing. The G-FINDER survey of neglected diseases R&D found that the biopharmaceutical industry was the third largest provider of R&D funding in 2008, after the U.S. government and the Bill & Melinda Gates Foundation [3]. Much of the research is done through partnerships. For example, in 2009 Merck entered into an agreement with DNDi (Drugs for Neglected Diseases initiative; http://www.dndi.org/press-releases/2009/480-merck-a-co-inc-and-drugs-for-neglected-diseases-initiative-collaborate-to-find-treatments-.html) to support discovery and development of improved treatments for neglected tropical diseases.
- ***Developing new approaches to help foster access to medicines in emerging and least developed countries*** **.** In the past decade, many pharmaceutical companies have implemented innovative differential pricing frameworks that reflect levels of economic development, payer category, and disease burden. Merck was one of the first in the industry to have a formal policy of differential pricing for its antiretroviral medicines and newest vaccines (http://www.merck.com/corporate-responsibility/access/access-developing-emerging/approach.html), and has since taken the approach of differentiating its pricing based upon a particular country's level of economic development for many of its newer products for chronic disease, such as its medicine for type 2 diabetes. Another tool to improve access is voluntary licensing. Many companies have granted voluntary licenses to generic companies to improve access to medicines in resource-limited countries. The industry has also developed philanthropic partnerships to help foster access, such as the MECTIZAN Donation Program (http://www.mectizan.org/history), through which Merck works with the World Health Organization, the World Bank, ministries of health, nongovernmental organizations, and local communities to provide medicine free of charge to more than 80 million people annually to treat river blindness.
- ***Supporting efforts beyond drug development and delivery, including helping States build health care capacity and strengthen health systems*** **.** Grave systemic obstacles in many countries hinder progress toward achievement of the right to health. Although clearly a State responsibility to address, many pharmaceutical companies have undertaken a variety of programs and partnerships to support State efforts to strengthen national health care systems. Merck has worked, for example, with the government of Botswana (http://www.merck.com/corporate-responsibility/access/access-hiv-aids/access-hiv-aids-ACHAP-botswana/home.html) and the Bill & Melinda Gates Foundation for the past ten years to develop and implement a comprehensive approach to the HIV/AIDS pandemic that addresses treatment, prevention, care, and support. Botswana's PMTCT and treatment programs have resulted in halving the mortality rate in adults in that country between 1980 and 2007 [4]. PEPFAR also reports a dramatic reduction of mother-to-child transmission in Botswana, leading to an 80% reduction in new infections among children (http://www.pepfar.gov/press/79674.htm). Such programs are not unique to Merck; a recent study by the International Federation of Pharmaceutical Manufacturers & Associations reported that there are 213 industry partnership programs to help improve health in developing countries [5].

## What More Should Be Done to Realize the Right to Health?

While States must continue to bear primary responsibility for the realization of the right to health, its attainment requires a multi-stakeholder approach. Everyone has a role to play and everyone can do more.

Pharmaceutical companies could better assess their role and monitor their contributions to advancing the achievement of the right to health. To this end, more companies could document their contributions to the health-related UN Millennium Development Goals. Several companies are working with the Danish Institute for Human Rights to develop a sector-specific human rights assessment tool that will examine core activities—including research and development, registration, pricing, licensing, and donations—that are fundamental to improving access both to medicines and health care overall. Such efforts should continue. Increasing transparency is another area where notable progress has been made by the industry but opportunities for improvement still exist.

Progress is possible, but it will require the international community coming together to support States' obligations and committing to meaningful dialogue on the true barriers and drivers for realizing the right to health.
